# Supplementary figures and images for: The NOSTRA model: Coherent estimation of infection sources in the case of possible nosocomial transmission
Source: PLoS Comput Biol. 2025 Apr 21;21(4):e1012949. doi: 10.1371/journal.pcbi.1012949 (PMC12121921; doi:10.1371/journal.pcbi.1012949)

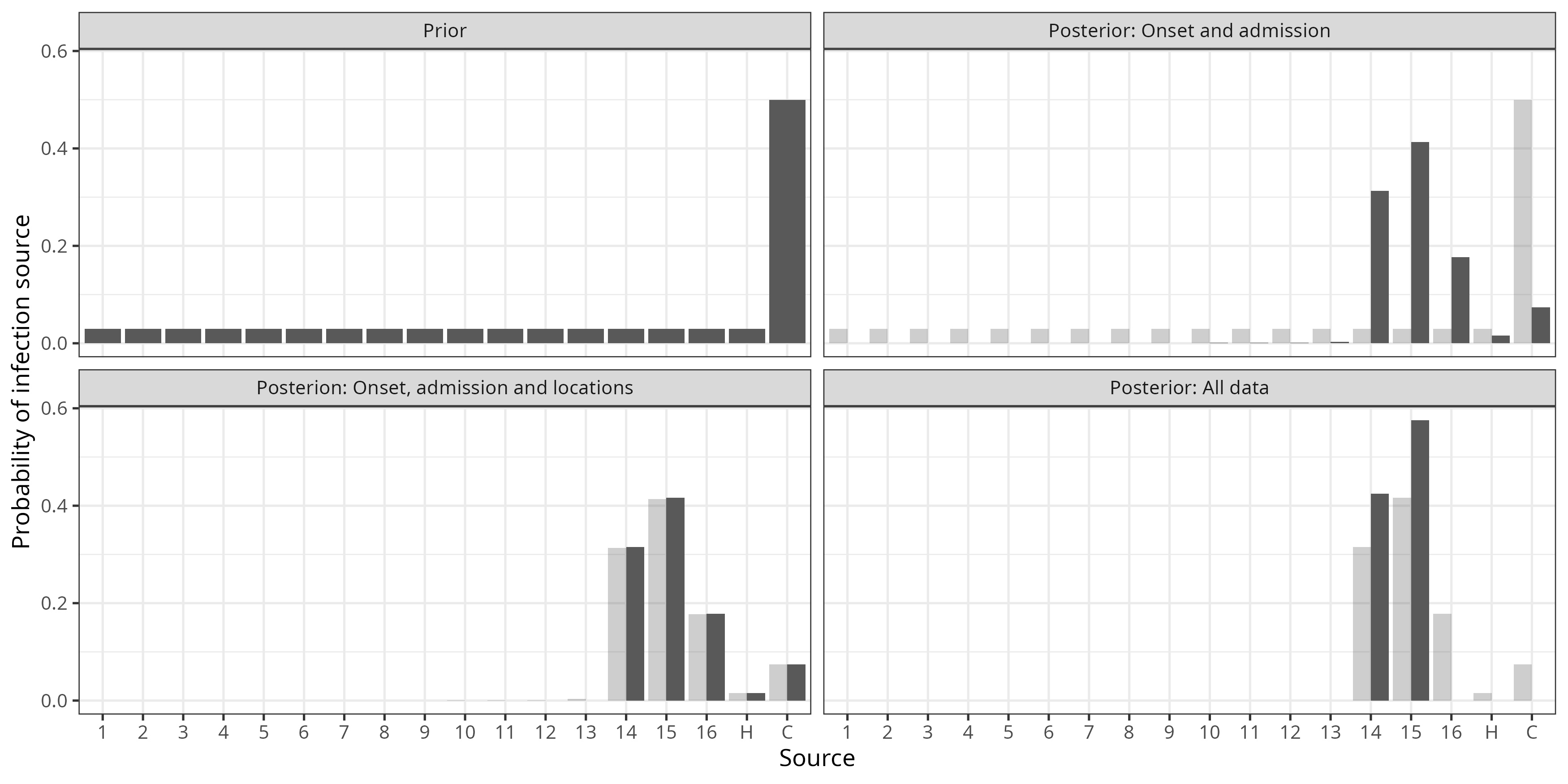

Supplement: S1 Fig — This figure shows an example NOSTRA run from one simulated individual from the simulation analyses. Potential candidate infectors for individual are labelled 1 to 16. The high of each bar corresponds to the posterior mass placed on that infection source. The top left panel shows the prior probabilities of each infection source. The top right panel shows the posterior probabilities of each infection source after admission and onset times are added (dark) and the prior probabilities of each infection source (light). The bottom left panel shows the posterior probabilities of each infection source after admission times, onset times, and location information are added (dark) and the posterior probabilities of each infection source after admission and onset times are added (light). The bottom right panel shows the posterior probabilities of each infection source after all data are added (dark) and the posterior probabilities of each infection source after admission times, onset times, and location information are added (light). (TIFF) [file pcbi.1012949.s002.tiff]
