# Supplementary material for: The NOSTRA model: Coherent estimation of infection sources in the case of possible nosocomial transmission
Source: PLoS Comput Biol. 2025 Apr 21;21(4):e1012949. doi: 10.1371/journal.pcbi.1012949 (PMC12121921; doi:10.1371/journal.pcbi.1012949)
Supplement: Attachment — Submitted filename: NOSTRA_review_response.docx [file pcbi.1012949.s003.docx]

Apologies for the multiple delays in providing a decision on this manuscript. As you will see from the reviewer comments, there are some common themes that will be important to address in your revised manuscript. In particular, both Reviewers 1 and 3 note the need to validate the model against simulated data. This is also important from a reproducibility standpoint, as it is noted that the original data cannot be shared due to privacy concerns, but a simulated dataset can and should be provided to allow for model testing. The reviewers also note the importance of being clearer about the limitations of the approach, e.g. that it only applies to acute infections.

We thank the reviewers for their largely positive comments. In response to your suggestions we have added a simulation study, which we believe as significantly improved the manuscript, as well as made changes throughout the text. Each of your comments are addressed explicitly below.

Reviewer's Responses to Questions

**Comments to the Authors:
Please note here if the review is uploaded as an attachment.**

Reviewer #1: This manuscript presents NOSTRA, a new model designed to estimate the probability of detecting a hospital acquired infection and the probabilities of the source being within a set of candidate individuals. Calculating these probabilities is set up as a Bayesian inference problem and thus requires specifying a prior and generates a posterior probability distribution. The authors demonstrate the utility of their method using previously published data collected during the early stages of the COVID-19 pandemic. They use data from one out of five hospital wards in the published dataset where patients were tested for COVID through targeted patient screening in wards with detected hospital-onset outbreaks. Data on patients includes movement, the genetic sequence of their infecting pathogen, their times of admission to hospital and symptom onset, and the movements and onset times of a set of n candidate infector individuals in the hospital. Some patients have associated genetic data while other patients are missing this information. The results demonstrate that probabilities of the infection source can be estimated, and that adding data sources improves their estimates. The authors are clear about assumptions and when NOSTRA should be and should not be used, and clearly define the purpose of their tool.

Critical Comment:

Overall, I believe the authors have developed a useful tool on solid mathematical and statistical foundations. My main concern/critique, which needs to be addressed in the revision, is that this paper completely lacks any analysis or demonstration that the uncertainty or biases around the estimates produced by the model are reasonable. I do not think the manuscript should be published until metrics of uncertainty are included. I think the most robust way to go about testing this new method would be to create simulated datasets with known infection origins and infectors and determine how often your model can predict the correct designation with a high probability. It would also be helpful if the authors could present credible intervals around each of their probability estimates and show how these intervals change if the patient has genetic data associated with their test. If there are too many estimates to put in the main text, the authors could add a supplement to their paper to house these results. Figures depicting uncertainty would greatly strengthen the paper.

We thank the reviewer for this comment and agree that this was an important problem with the original submission. We have now performed a full analysis on simulated data which has been added to the results section, and comment on the strengths and limitations of the model from this context throughout the discussion section. We hope that this resolves this issue. As for the discussion of credible intervals, there actually are none, as the analytical posterior distribution is Categorical, so the uncertainty is between classes rather than in the estimated probability for each class.

Major Comments:

The Bayesian Analysis: Inference section does not offer enough detail. I think it would be helpful to any future user of your method to describe the algorithm you used to sample from your posterior (or suggest one) or state you are calculating this distribution directly. It is not clear what you are doing unless you look at your code.

Thank you for this note. We have added that we are calculating the distribution directly in the methods section and noted in the discussion section that a large number of the choices we made in the modelling process are to maintain the analyticity of the posterior distribution, which allows this direct calculation.

The authors should offer guidance on how to interpret the probabilities. Is there a cutoff the authors recommend to designate, for example, if an infection is either nosocomial or not?

We have addressed this in a new paragraph in the discussion, but in summary our answer is that there isn’t a right answer. See below:

NOSTRA provides probabilities, not dichotomised answers to the question of whether an infection is nosocomial. Thus, an important question that we have not attempted to answer in this work is how healthcare workers should interpret these probabilities for action. We believe that labelling a particular infection as "nosocomial" (or otherwise) on the basis of the model probability is, in effect, a policy decision. As such, this should be taken with reference to the potential costs and benefits of any action that would be taken in response to that decision, and how these would change if the classification is wrong. Therefore, it is important that thresholds for labelling be determined by individuals embedded within healthcare systems who are aware of the economic consequences of the decision for that particular system, and it is implausible that any one-size-fits-all approach will be appropriate.

Overall, figures and figure captions need to be more informative:
First, I think it would be beneficial to have at least one figure that demonstrates the uncertainty around your estimates.

Regarding the credible intervals: our Bayesian model aims to estimate the true infection source for each individual. The "parameters" of that model are the categorical variables indicating the infection source. Therefore uncertainty about those parameters is described by the probability that the true source is one of a list of candidates. Hence the concept of uncertainty / credible intervals around those probabilities does not make sense in this context.

Figure 1 – Why is hospital and nosocomial included on the x-axis? What is the difference between hospital and nosocomial? Also, what is the “full model”? Please remind the reader in the caption what the figure represents.

We have modified the caption, but the “Hospital” compartment represents hospital sourced infections that are unlikely to be from one of the explicit infection candidates within the hospital. We have renamed this “Other hospital source” in the figure. Thus the probability that the infection is nosocomial is the sum of all the explicit candidates and the “Hospital” compartment. Hopefully the adjustments to the figure and caption have made this clearer. Here is the new caption, changes italicised:

“A visualisation of the output of the model run *with all available data* applied to the full CUH Ward A dataset. Each row corresponds to a candidate individual, each column, except the last, to a potential infection source. *Sources starting with "CAMP" are identified patients within the hospital. The "Hospital" source represents all unidentified sources within the hospital. The "Community" source is all sources outside the hospital.* Cells are coloured by the posterior probability of that infection source. The last column shows the posterior nosocomiality probability, *which is 1-P(Community|Data)*.”

Figure 2 – It would be helpful to have the panels labeled (e.g. A, B, etc.) and describe specifically what each panel represents. It seems like the very last panel in this figure is different (e.g. it is not a measure of change in probability). What results are shown in the last panel, and should it be a separate figure?

We have modified Fig 2 to add labels, and changed the figure legend to better explain why one of the panels is not a change in probability. The new text is below:

***The impact of adding data sources on the assessed probability of each infection source.*** *Panel a. shows the posterior probabilities of each infection source when only symptom onsets and admission times are provided to NOSTRA. The other panels explore the impact on the posterior probabilities as different data is added. Panel b. shows the change in posterior probability from the estimates in panel a. when location information is added. Panel c. shows the change in posterior probability from the estimates in panel a. when genetic information about the pathogen is added. Panel d. shows the change in posterior probability from the model including onset times, admission times and patient locations, when genetic information about the pathogen is added. Panel e. shows the change in posterior probability from the model including onset times, admission times and genetic information about the pathogen, when patient locations are added.*

I do not think the authors adequately justify the importance of understanding the origins of infections in health care settings. I think this is a missed opportunity. For example, in line 6 the authors state: “These two scenarios have very different implications for decision making.” But then only follow this statement by explaining one of the scenarios (ward closure), but not both. I think additional impacts of identifying if an infection is nosocomial or not and the transmission chain are more than just ward closure. This is explained in line 46 with regard to COVID, but this seems like it could be generally applicable to many different types of infections (e.g. bacterial infections). I would like to see the authors add more detail in the first paragraph of the introduction explaining why this is generally important. (This could also be mentioned in the conclusion).

We have modified the first paragraph of the introduction to better set the scene:

Nosocomial infections are an important issue facing health systems across the world, impacting both patient survival and their willingness to access healthcare. However, when an infection is found in a patient in a healthcare setting it is often unclear whether the infection was genuinely acquired in hospital or just detected there. These two scenarios have very different implications for decision making. High levels of nosocomial infections may require the stepping up of infection control responses both at the ward level, and across the whole hospital to protect patient health. The precise required response may be idiosyncratic, as the ultimate causes of nosocomial infections differ dramatically across pathogens and facilities. For example, poorly ventilated wards may be susceptible to frequent nosocomial respiratory infections, while wards containing particularly susceptible patient groups may be at risk of high rates of nosocomial infection across pathogen groups. The ideal responses to these two cases will be very different, and the set of available responses will be limited by the economic environment the facility is working in. But while the responses between scenarios and facilities will differ, in order to know whether they are necessary at all, it is first necessary to determine whether the infections that are being seen truly are healthcare-associated. Given this, methods for assessing both whether an infection is nosocomial, and if it is, whether it is part of a large hospital outbreak, are necessary for coherent decision making in infection control.

I am unable to run any of the code in the NOSTRA-model repository. If the data cannot be made available, the authors should produce a simulated dataset that a user can use to test the code. Additionally, there is no information about the repository in the Readme file. The code needs to be described in the Readme file in the repository.

We have modified the NOSTRA-model github to have full instructions in the README to replicate the simulation study, with the data required to replicate the study being downloadable from <https://doi.org/10.6084/m9.figshare.27172758>. Please be aware that the simulation study analysis code takes about 4 days to run on our system, so if you attempt to replicate this yourself expect a runtime of this order of magnitude.

Minor Comments:

Abstract - This is confusing: “example dataset from a real dataset”. It is not clear until reading the methods what the authors mean by “example dataset.”

We have modified this sentence to:

*We illustrate this model on an example dataset from a real hospital showing both its output and how the impact of the different data sources on the assessed probabilities are contingent on what other data has been included in the model, and validate the calibration of the predictions in a large simulation study.*

Line 93 - The use of viral sequences in line 93 seems abrupt. For readers who are not familiar with genomic sequences, it would be helpful to expand and introduce this method with a bit more detail.

We added the following text to provide some extra background:

These sequences represent the string of nucleic acids that make up the viral genome, and differences between them can be informative on how the viral populations within different patients are related to one another. The whole viral population in a patient is summarised as a "consensus sequence", which can be viewed as the average viral genome in that patient.

Consider moving some of the mathematical details about the likelihood section to an online supplement.

We considered this suggestion, and there was disagreement among the authors about whether to proceed. Eventually we decided that, as this is a methods paper, and the structure of the model is the most fundamental part of the manuscript, it is important to keep the details of the likelihood front and centre in the final presentation of the paper.

Reviewer #2: Thank you for the opportunity to review the manuscript by Pascall and colleagues that describes a Bayesian model that estimates whether an infection detected in hospital is of nosocomial origin, as well as the likelihood of being derived from a proposed group of source patients. The manuscript is well written and addresses a frequently encountered, but challenging problem of determining source of acquisition for infections detected in hospitalised patients. In practice, Infection Prevention & Control interventions often take a conservative and sometimes excessive approach when applied to outbreak control, though this is due to the difficulty in identifying the source in each case. In this manuscript, the authors provide a glimpse of what may be possible to glean from detailed genomic and epidemiological data and I believe would be of interest to healthcare administrators and those working in Infection Prevention & Control.

It should be noted that I have reviewed the manuscript from a clinician perspective as although I have some rudimentary knowledge of modelling, I do not have the detailed mathematical background to review the model in detail. I have included some comments below intended to help improve the readability and interpretation of the manuscript from a clinician perspective.

1. It would be helpful if Figures 1 and 2 could be presented in the same orientation to aid interpretation.

We have regenerated Figure 2 in a portrait formation.

2. Could you please define the terms "nosocomial", "community" and "hospital", as used in the output of your model? The definition of nosocomial usually includes transmission from any individual in the healthcare environment, not just from the set of candidate individuals i.e. {A,H}. However, I understand your rationale to separate these as infections from "known positives" (I think what you have labelled "nosocomial") and from unrecognised sources in the hospital environment (I presume what you have labelled "hospital").

We have added a discussion of the precise definition of these terms at the bottom of the methods section. But, in short, nosocomial is any infection from a source in the set {A,H}, with the split beyond that being into identified candidate sources, and unrecognised sources.

Here’s the added text:

*This gives us the posterior probability of all the infection sources being the source of the infection. The community probability, P(S=C|X), is the probability the infection was obtained outside the medical facility. The nosocomiality probability is 1-P(S=C|X), that is, the sum of all the non-community probabilities. The probability of the hospital compartment, P(S=H|X), is the probability that the infection came from a source within the medical facility which had not been* a priori *identified by the medical staff.*

3. Transmission from individuals in the hospital that are outside of the set of candidate individuals are also relevant to healthcare-associated outbreaks, and could be from staff, visitors, or other unrecognised patient cases, each with a different outbreak control intervention. This is alluded to in the discussion, but consider expanding the discussion on the potential for monitoring number of healthcare-associated cases that are related to "known positives" vs those from "unrecognised sources" in the hospital.

We added another section alluding to this possibility. At the moment, we would not recommend such a use for the model, as it wasn’t explicitly designed for monitoring, and we are not sure how well it would perform at that role.

*This does mean that, in cases where the "Hospital" source is identified as the most probable, it likely does indicate that the detected infection genuinely is unrelated to anything in the candidate set. Therefore, while NOSTRA is not designed for this, with appropriately designed candidate sets (i.e. excluding a class of interested), NOSTRA could potentially be used to explore whether different classes of individuals are involved in within hospital outbreaks. However, we would advise further validation studies be performed before this were attempted.*

4. Consider a box listing key caveats of implementing NOSTRA e.g.
- requires infection with short, well-defined incubation periods and generation times (given the dependence on this to infer nosocomiality/hospital-acquisition)
- requires a reasonable proportion of the samples in the hospital to be sequenced
- better suited to pathogens without significant within-host variation

We have added this as Table 2.

Reviewer #3: The manuscript by Pascall et al. describes a new model (NOSTRA) for reconstruction of transmission of infections in the hospital environment.

The NOSTRA model builds upon two existing models developed by the group: the A2B model that determines who infected whom in hospital outbreaks, and the HOCI model that assesses the probability that an infection was nosocomial. The authors report that the originality of this model is that it would be the first to simultaneously assesses whether an infection is nosocomial in origin (versus community-acquired) and who the likely infector is.

This research question addressed by the authors is both important and relevant, for the modelling community but also for infection prevention and control professionals working in hospitals. The authors should be lauded for the thoroughness of their approach and the significant amount of work that was put into this. Additional strengths of the manuscript include the use of ward location data for patients, which are, to the best of our knowledge, not used in publicly available approaches. Finally, the manuscript is well-written and easy to follow conceptually.

Despite these strengths, there are major limitations that we would like to highlight.

The scope of this approach is not useful for all nosocomial infections – it would only be useful for acute viral respiratory infections. In the case of bacterial infections, even in outbreak situations, there exists the problem of asymptomatic carriage which is not accounted for in the model. Please ensure that this is made clear in the title and main text, or explain how asymptomatic carriage (with or without subsequent infection) are accounted for.

The question of asymptomatic carriage is interesting, in that it doesn’t really impact the model too much, given our strong assumptions about missingness. (A much more problematic case is indirect transmission via the environment, which NOSTRA cannot handle at all, and has been added as a caveat in the table in the discussion.) As NOSTRA is robust to missing data, as long as the focal individual isn’t asymptomatic, the model can just be run absent onset times for identified candidates who are asymptomatic. The genetics of the isolates from those asymptomatic candidates will then still inform the likelihood. Or they can be left out and they will just be implicitly included in the “H” source as usual.

We added the follow text to the discussion on this topic:

A type of missing data that is worth commenting on specifically is asymptomatic carriage. In the case of asymptomatic carriage symptom onset times will be unobserved (and, indeed, undefined). While we did this for illustrative purposes with the CUH data, we strongly advise that, in practice, NOSTRA should not be applied to focal individuals who are asymptomatic. This is because there is no well-defined distribution that describes the waiting time between admission and detection for asymptomatic individuals. It is entirely a function of the testing strategy applied by the healthcare facility. This does not mean that NOSTRA is inapplicable to pathogens where asymptomatic carriage is an important factor in the epidemiological dynamics, as long as the focal individual is symptomatic. If one is willing to accept the missing completely at random assumption, candidate infectors who are asymptomatic can be included in the model with their onset times as missing data, this allows the genetics of their pathogen to still inform the likelihood. If one is not willing to make that missingness assumption, these individuals can simply be ignored, which is equivalent to including them in the Hospital source.

The premise that simultaneous determination of “nosocomiality” and source attribution would be better than sequential assessment is not in itself intuitive. Can the authors please expand on what the added value of this approach would be? Also, it would be interesting to perform a comparison of the characteristics of the NOSTRA model and other existing approaches that could be used to address the same problem.

In the new Figure 3, we show the dramatic gain in predictive skill that is acquired by the joint modelling approach as opposed to treating the problem of nosocomiality separately to that of source attribution. This is the “NOSTRA – Candidates” vs. “NOSTRA - No candidates” contrast. The information gain here is because, when the nosocomiality status of an infection is uncertain based on the admission to detection time, the presence of infections genetically similar to the focal infection within the hospital is a very useful indicator that the infection may well have been acquired within hospital.

We did not compare NOSTRA to other methods to answer this question, because attempting to do so would have required skill with models that the authors do not possess, and we did not want to compare NOSTRA (which we understand how to make perform well) to a suboptimal implementation of others’ models. Instead, during our simulations we compare NOSTRA to the series of references outlined in the text, which includes the 96hr heuristic rule.

While we agree with the authors’ premise that the definitions of nosocomial infection are somewhat arbitrary (and perhaps most useful for surveillance purposes), we are left wanting more regarding how useful this model would be in practice. Presumably, there are some cases where the attribution of “nosocomiality” are straightforward. For what proportion of cases do the authors expect there to be a benefit?

In the process of adding our simulation study, we’ve added a discussion of “difficult” cases. In short the argument is that nosocomial assessment is easy when the symptom onset time-admission time difference is inconsistent with infection in the hospital or community. When exactly this applies will vary with the incubation time of the pathogen under study. In our SARS-CoV-2 simulation analysis, we see that the difficult cases are occurring when symptoms appear 2 or 3 days post admission, and this is when models would be expected to be most useful. But this time window will not generalise to other pathogens with different incubation times, and in general, we suspect that the greater the variance of the generation time distribution of the pathogen under study, the more useful models will be.

A major limitation is the lack of assessment of the model’s performance in a simulated outbreak, where the “ground truth” is known. A comprehensive simulation study is essential to understand the model's behaviour under different conditions, as commonly done with novel outbreak reconstruction tools. Evaluation typically involves diverse conditions like population sizes, R0 values, sampling coverage, mutation rates, and generation time/serial interval distributions.

We had added a simulation study to the paper. Due to the complexities of hospital outbreaks, we could not build our own simulation model in the time allotted for this review, so we instead used a previously published agent-based hospital simulation model built for SARS-CoV-2. This model has a set of 20 previously validated parameter sets for running hospital infection simulations. We then simulated the mutation process on top of the transmission trees generated from this model. We tested NOSTRA model under simulations from each of these parameter sets, at three different background prevalence levels. Hopefully this is detailed enough to address this comment.

Another limitation lies in the the fact that healthcare workers do not seem to be included in the model. It is well-established that healthcare workers are key drivers of nosocomial outbreaks of acute respiratory viral infections.

While we have not explicitly accounted for healthcare workers in the model, they are included implicitly. There is no reason, for example, that one of the identified candidate individuals could not be a healthcare worker . Also, the “H” source includes any healthcare workers who are not explicitly identified as candidate individuals. We have added a note to this effect in the discussion.

We disagree with the authors’ premise that “the appropriate infection control response depends on whether an infection truly is nosocomial”. The unexpected occurrence of a respiratory viral infection in a patient who was not symptomatic of that infection on admission would trigger the same investigations and response whether the infection is acquired in the hospital or the community.

We have changed this in response to another reviewers comment, but it also addresses this one. Our original statement was not meant to imply that the investigations may differ, but that the correct response in the face of frequent nosocomial infections versus frequent imported infections is different.

Additional comments
• It may be worth assessing the model's robustness to changes in population size, as suggested in a related study focusing on detecting imports from a genetic coalescent model (<https://www.ncbi.nlm.nih.gov/pmc/articles/PMC9805578/>).

We agree that this would have been a valuable addition to the study, especially as we had already called out our coalescent assumptions as a potential weakness, however, it was not practical in the time available for review.

• How are asymptomatic cases handled?

We handle asymptomatic cases in a slightly ad hoc way, and use their detection date in place of their symptom onset date in the same modelling framework. This isn’t ideal, but we couldn’t think of a reasonable way of defining what the distribution of time from infection to detection would look like for symptomatic patients, as it would be largely a function of testing strategy.

• How does not accounting for missing links/generations affect the inference about “nosocomiality” or the source?

Missing links actually aren’t a huge issue for the assessment of nosocomiality. If any individuals in the same transmission chain as the focal individual are detected, they should drive up the probability of nosocomiality even if the true source is missed. It’s a much larger problem for source attribution. Technically speaking, if the source is a missing link but other individuals in the transmission tree are detected the model should put the posterior probability on the “H” compartment. In practice this doesn’t happen because the data from an individual two generations back looks almost exactly the same as the data from an individual one generation back, especially if the are all on the same ward simultaneously. So NOSTRA ends up giving high posterior probability to the individual two generations back. We have added a large section in the discussion about this phenomena and how the source probabilities should be interpreted considering it.

• “Waiting time” (line 147) should be replaced by “incubation period”.

Thanks. Fixed.
